# Tertiary lymphoid organs are associated with the progression of kidney damage and regulated by interleukin-17A

**DOI:** 10.7150/thno.48624

**Published:** 2021-01-01

**Authors:** Ran Luo, Yichun Cheng, Dan Chang, Tingting Liu, Liu Liu, Guangchang Pei, Nanhui Zhang, Zufeng Wang, Kanglin Guo, Wei Chen, Ming Li, Li Fan, Chunxiu Zhang, Yueqiang Li, Wei Dai, Meiying Zuo, Yulin Xu, Ying Yao, Shuwang Ge, Gang Xu

**Affiliations:** 1 Department of Nephrology, Tongji Hospital, Tongji Medical College, Huazhong University of Science and Technology, Wuhan, China.; 2 Department of Nephrology, The First Affiliated Hospital, Sun Yat-sen University, Guangzhou, China.

**Keywords:** Tertiary lymphoid organs, kidney damage, IL-17A, progression, inflammation

## Abstract

**Background:** Tertiary lymphoid organs (TLOs) occur after multiple chronic kidney injuries. interleukin-17A (IL-17A) has been reported to associate with the development of TLOs in inflammatory diseases. However, regulation of the renal TLOs and its clinical significance to the pathogenesis of chronic kidney injury are unknown.

**Methods:** To evaluate the clinical significance and regulation of renal TLOs, we analyzed the progression of patients with kidney damage based on the existence and absence of TLOs in a larger multicenter cohort. We also blocked the recruitment of lymphocyte cells into the kidney by FTY720 (fingolimod) *in vivo*. Besides, we used aged IL-17A genetic knocked out mice and IL-17A-neutralizing antibody to explore the role of IL-17A in renal TLOs formation.

**Results**: We demonstrated that renal TLOs of IgA nephropathy patients were associated with disease severity and were independent risk factors for renal progression after adjustment for age, sex, mean arterial pressure, proteinuria and, baseline eGFR and MEST-C score, especially in the early stage. Plasma levels of TLO-related chemokines CXCL13, CCL19, and CCL21 were higher in patients with renal TLOs. Inhibiting the formation of renal TLOs by FTY720 could reduce the intrarenal inflammation and fibrosis, and early intervention was found to be more effective. IL-17A was increased in renal TLOs models, and genetic depletion of IL-17A or treatment with anti-IL-17A antibody resulted in a marked reduction of the TLOs formation as well as alleviation of renal inflammation and fibrosis.

**Conclusion:** These results indicate that TLOs are associated with the progression of kidney damage and regulated by IL-17A and may be effective targets for the treatment of kidney damage.

## Introduction

Previous clinical studies and animal experiments have shown that interstitial inflammation is involved in the pathophysiological process of chronic kidney injury 1-3. Infiltration of various inflammatory cells, such as T cells, B cells, macrophages and dendritic cells; is observed in chronic kidney injury 2,4. However, the underlying mechanism of the immune cell infiltrate composition to the progression of chronic kidney injury is not fully understood. In addition, there is no effective treatment to inhibit inflammation and alleviate the progression of kidney disease.

Tertiary lymphoid organs (TLOs) are ectopic lymphoid tissue in non-lymphoid organs 5. A well-organized TLO is composed of T cells, B cells, follicular dendritic cells, fibroblastic reticular cells (FRCs), high endothelial venules (HEVs) and lymphatic vessels (LV). TLOs exist in patients with cancer 6, allograft rejection 7, inflammatory conditions 8 or autoimmunity 9; however, the effect of TLOs on the prognosis of different diseases is contradictory 6. Increasing research on this topic has revealed a positive association between tumor-associated TLOs and a favorable clinical outcome for patients with cancer 6. It was reported that TLOs with evidence of local humoral immune response are present in chronic rejection 10, and the density of TLOs was an independent predictor of a more aggressive disease phenotype of Sjogren syndrome 11. Our previous studies have reported the presence of renal TLOs in patients with IgA nephropathy (IgAN) 12, but the relationship between renal TLOs and prognosis has not been demonstrated in large clinical cohorts. Furthermore, a non-invasive predictive biomarker is needed to reflect the development of renal TLOs.

The development of TLOs is initiated by specialized immune cells called 'lymphoid tissue inducer' (LTi) cells which accumulate at the site of inflammation and interact with stromal mesenchymal cells called 'lymphoid tissue organizing' (LTo) cells via binding of LTα and LTβ on LTi cells with LTβR on LTo cells 13. This leads to the release of chemokines CCL19, CCL21, and CXCL13 from stromal cells 14,15. These chemokines mediate further immune cell attraction and trafficking within the forming TLOs. The critical role of IL-17A in the pathogenesis of multiple kidney diseases has been reported before 16. Increasing levels of circulating IL-17A are described in patients with chronic kidney disease 17, lupus nephritis (LN) 18, membranous nephropathy (MN) 19, and Henoch-Schönlein purpura nephritis 20. IL-17A gene knockout or IL-17A neutralizing antibody blockade alleviate renal injury and decrease renal inflammation in mice models of septic acute kidney injury 21, LN 22, and diabetic nephropathy 23. In addition, Th17 cells share some common developmental factors, transcription factors, and cell surface markers with LTi cells 24. IL-17A has been reported to be associated with the development of TLOs, which are induced by an experimental autoimmune encephalomyelitis (EAE) mouse model of multiple sclerosis 25 or by lipopolysaccharide-induced pulmonary inflammation 26. However, the role of IL-17A in renal TLOs development has been rarely reported.

To evaluate the clinical significance of renal TLOs, we analyzed the progression of IgAN patients on the basis of the existence and absence of TLOs in a larger multicenter cohort. We established three kidney injury models using aged mice, in which multiple renal TLOs were developed. To further research the regulation of TLOs, we blocked recruitment of lymphocyte cells into the kidney by FTY720. We used aged IL-17A genetic knocked out mice and IL-17A-neutralizing antibody to investigate the role of IL-17A in renal TLOs formation.

## Methods

### Participants

#### Tongji cohort

A cohort of 847 patients with renal biopsy proved IgAN were enrolled at Tongji Hospital from January 2012 to November 2016. We excluded the patients age < 18 year (N = 20), with end-stage renal disease (ESRD) at the time of biopsy (N = 4), with missing baseline data (N = 102) and lost to follow-up (N = 172). Finally, 549 patients (42.3% men; mean age 35 ± 11 years) were included.

A cohort of 100 patients (12.0% men; mean age 32 ± 10 years) with renal biopsy proved LN were enrolled at Tongji Hospital from January 2011 to November 2016.

A cohort of 127 patients (60.6% men; mean age 44 ± 15 years) with renal biopsy proved MN were enrolled at Tongji Hospital from January 2012 to December 2016.

A cohort of 49 patients (55.1% men; mean age 35 ± 16 years) with renal biopsy proved minimal change disease (MCD) were enrolled at Tongji Hospital from January 2009 to December 2017.

#### Sun Yat-sen cohort

A cohort of 995 patients with IgAN were enrolled at The First Affiliated Hospital of Sun Yat-sen University from January 2000 to May 2010. We excluded the patients age < 18 year (N = 55), with ESRD at the time of biopsy (N = 43), with missing baseline data (N = 59) and lost to follow-up (N = 343). Finally, 495 patients (39.4% men; mean age 32 ± 10 years) with were included.

This study was approved by the Ethical Committee of Huazhong University of Science and Technology (no. TJ-IRB20181108) and the Ethical Committee of Sun Yat-sen University (Lunshen 2016012). All procedures performed in this study were in accordance with the ethical standards of the institutional and/or national research committee and with the 1964 Helsinki Declaration and its later amendments or comparable ethical standards. Human tissue and blood samples were obtained from patients with written informed consent. Combined event was defined as 50% decline of eGFR or end-stage renal disease.

### Chemokine measurements by Luminex assay

The concentration of CXCL13, CCL19 and CCL21 in plasma of randomly selected 73 IgAN patients from Tongji Cohort was analyzed by the multiplex Luminex™ assay from R&D Systems according to the manufacturer's protocol. Blood samples were collected at the time of renal biopsy and centrifuged for 5 min at 2,000 *g* immediately, and then stored in aliquots at -80 °C until assayed.

### Immunohistochemistry and immunofluorescence

Formalin-fixed and paraffin-embedded kidneys were cut into 4 μm sections. Antigen retrieval was performed by citrate buffer (pH 6.0) for 20 min after deparaffinization and rehydration. For immunohistochemistry staining, renal tissue sections of 53 IgAN patients which were randomly selected from Tongji cohort were blocked with 10% H2O2 for endogenous peroxidase for 15 min and blocked with 5% serum for secondary antibodies for 30 min at room temperature, and then incubated overnight at 4 °C with primary antibodies against CD4 (EP204, Gene Tech), CD8 (SP16, Maxim-bio), and CD20 (L26, Gene Tech). Tissue sections were treated with horseradish peroxidase (HRP)-conjugated secondary antibodies followed with 3,3'-diaminobenzidine (DAB), which was used as an HRP-specific substrate. For immunofluorescence staining, tissue sections were blocked with 5% serum for secondary antibodies for 30 min at room temperature and then incubated overnight at 4 °C with primary antibodies against B220 (RA3-6B2, R&D Systems), CD3 (GB111337, Servicebio), CD45 (Abcam), CD21 (7G6, BD Biosciences), podoplanin (AF3244, R&D Systems), α-SMA (Abcam), LYVE-1 (AF2125, R&D Systems), IL-17A (sc-374218, Santa Cruz Biotechnology) and Ki67 (GB111499, Servicebio). Tissue sections were treated with appropriate fluorescence-labeled secondary antibodies. 4′,6-diamidino-2-phenylindole (DAPI) was used for nuclei staining. An Olympus microscope and DP73 camera were used for taking representative images.

### Compound administration

FTY720 (MedChemExpress) were prepared in phosphate-buffered saline (PBS) solution. FTY720 (0.5 mg/kg) or vehicle (PBS solution) was injected intraperitoneally one day before and daily after induction of renal ischemia until euthanized on day 45. Monoclonal anti-IL-17A antibody or IgG (Bio X Cell, West Lebanon, NH, 50 μg) were dissolved in 250 μL of PBS and injected intraperitoneally one day before and twice weekly after the induction of renal ischemia until euthanized.

### Real-time PCR

Total RNA was extracted from the kidneys with Trizol reagent according to the manufacturer's instructions (Invitrogen, USA). Template cDNA was obtained using a reverse transcription system kit (Takara, Japan). Quantitative polymerase chain reaction (PCR) was carried out using the SYBR master-mix (Takara, Japan). Each expression level was normalized by β-actin levels, which had been measured as an internal control. The designed primers are listed in Table S1.

### Flow cytometry

For FACS analysis of mouse renal cells, single-cell suspensions were stained with antibodies at 4 °C for 30 min. The following antibodies from Biolegend were used: BV421-conjugated Zombie (423114), APC/Cy7-conjugated anti-mouse CD45 antibody (103116), APC-conjugated anti-mouse CD3 antibody (100236), FITC-conjugated anti-mouse CD4 antibody (100406), PE/Cy7-conjugated anti-mouse CD8 antibody (100722), PE-conjugated anti-mouse B220 antibody (103208), PE/Cy7-conjugated anti-mouse CD31 antibody (102417), PE-conjugated anti-mouse GP38 antibody (127407).

### Genetic analysis

The data of single-nucleotide polymorphism (SNP) data were derived from a genome-wide association study, which included three independent samples of Han Chinese consisting of a total of 4,137 IgAN patients and 7,734 healthy controls 27. DNA was extracted from whole blood samples using standard methods. Genome-wide genotyping was performed using the Illumina Human 610-Quad BeadChip. In this study, we enrolled IgAN patients with complete baseline and follow-up data from the Sun Yat-sen cohort. Finally, a total of 918 were included in genetic analysis. The SNPs located in LTA 28-31, LTBR 32, CCL19 33,34 and CCL21 33,35 has been reported previously.

### Mice

C57BL/6J mice (age, 10-12 months; weight, 30-35 g) were purchased from Hua Fukang (Beijing, China). *Il17a^-/-^* mice (age, 10 months; weight, 30-35 g) were purchased from Biomodel Organism (Shanghai, China). All mice were maintained in specific pathogen-free conditions at Tongji Medical College of Huazhong University of Science and Technology. Mice were anesthetized and euthanized with 1% sodium pentobarbital solution (10 μL/g, Sigma, USA) by intraperitoneal injection. All animal experiments were approved by the Experimental Animal Ethics Committee of Huazhong University of Science and Technology.

### Kidney injury model

Mice were anesthetized with 1% sodium pentobarbital solution (0.01 mL/g body weight, Sigma, USA) by an intraperitoneal injection. For the unilateral ureteral obstruction (UUO) model, the left ureters of mice were exposed though a lateral incision and tied off with two 4.0 silk sutures. UUO mice were euthanized 14 days after the operations. Sham operated mice underwent an identical procedure but without ureteric ligation. For ischemic reperfusion injury (IRI) models, the left kidneys were exposed through flank incisions and were clamped with an atraumatic vascular clip for various durations of time (Roboz Surgical Instrument Co., Germany). IRI mice were euthanized 30 days or 45 days after the operations. Sham animals were subjected to a similar surgical procedure without clamping the left kidney pedicle. In the animal model, some mice showed hair loss or weight loss, which possibly related to the surgery. In the folic acid (FA) nephropathy model, mice received an intraperitoneal injection of 250 mg/kg of FA dissolved in 0.15 M of sodium bicarbonate (Sigma-Aldrich) on day 0 and were euthanized on day 21.

### Renal histochemistry

Kidneys were fixed in 4% neutral buffered formalin and embedded in paraffin. Periodic acid-Schiff (PAS) staining was performed to evaluate TLOs size, and Masson staining was performed to estimate renal fibrosis. TLOs size was defined as the total cumulative size of the TLOs in the renal cortex of the sample. Images that included TLOs were taken at the same size and resolution, and size of TLOs was measured using ImageJ software. For the quantitative analysis of renal fibrosis, the fibrotic area in Masson staining sections was quantified using ImageJ software in eight randomly selected microscopic fields and expressed as the percentage area, as previously reported 36,37. The fibrosis score for each sample was defined as the average ratios of fibrotic area to each microscopic field. Renal histologic lesions were graded according to MEST-C score 38.

### Transcriptome sequencing and bioinformatics analysis

Total RNA was isolated from kidney tissue and subjected to cDNA synthesis, fragmentation, adapter ligation, and PCR amplification. Sequencing was performed using the Illumina HiSeq platform. The sequencing reads were further processed with the determination of quality using the SOAPnuke tool. We mapped clean reads to mouse mm9 genome using HISAT (Hierarchical Indexing for Spliced Alignment of Transcripts). The fragments per kilobase million (FPKM) and differentially expressed genes (DEGs) were obtained using RSEM and DEGseq software, respectively (fold change ≥ 2 and adjusted *P* value ≤ 0.001). R package was used to conduct gene ontology (GO) and Kyoto Encyclopedia of Genes and Genomes (KEGG) analysis and construct protein-protein network interaction.

### Statistical analysis

Specific numbers of animals are expressed in each figure legend. Statistical analysis was carried out using SPSS 23.0 software (SPSS, USA) and GraphPad Prism version 6 software (Graph software, San Diego, CA). Categorical variables were summarized as percentages, normal distribution data were expressed as mean ± standard error of mean and abnormal distribution data were expressed as median with interquartile range. Correlations were evaluated using nonparametric Spearman's and parametric Pearson's correlation tests. We used Cox regression and Kaplan-Meier survival curves to analyze the association between renal TLOs and renal outcome, and the significance was determined by the log-rank test. We used the one-way ANOVA test, unpaired *t* test or Mann-Whitney test to evaluate *P* values as appropriate. Chi-square tests were used for categorical variables. n.s. > 0.05, *P < 0.05, **P < 0.01, ***P < 0.001.

## Results

### TLOs were found in the kidney of patients with kidney damage

**Figure 1:**
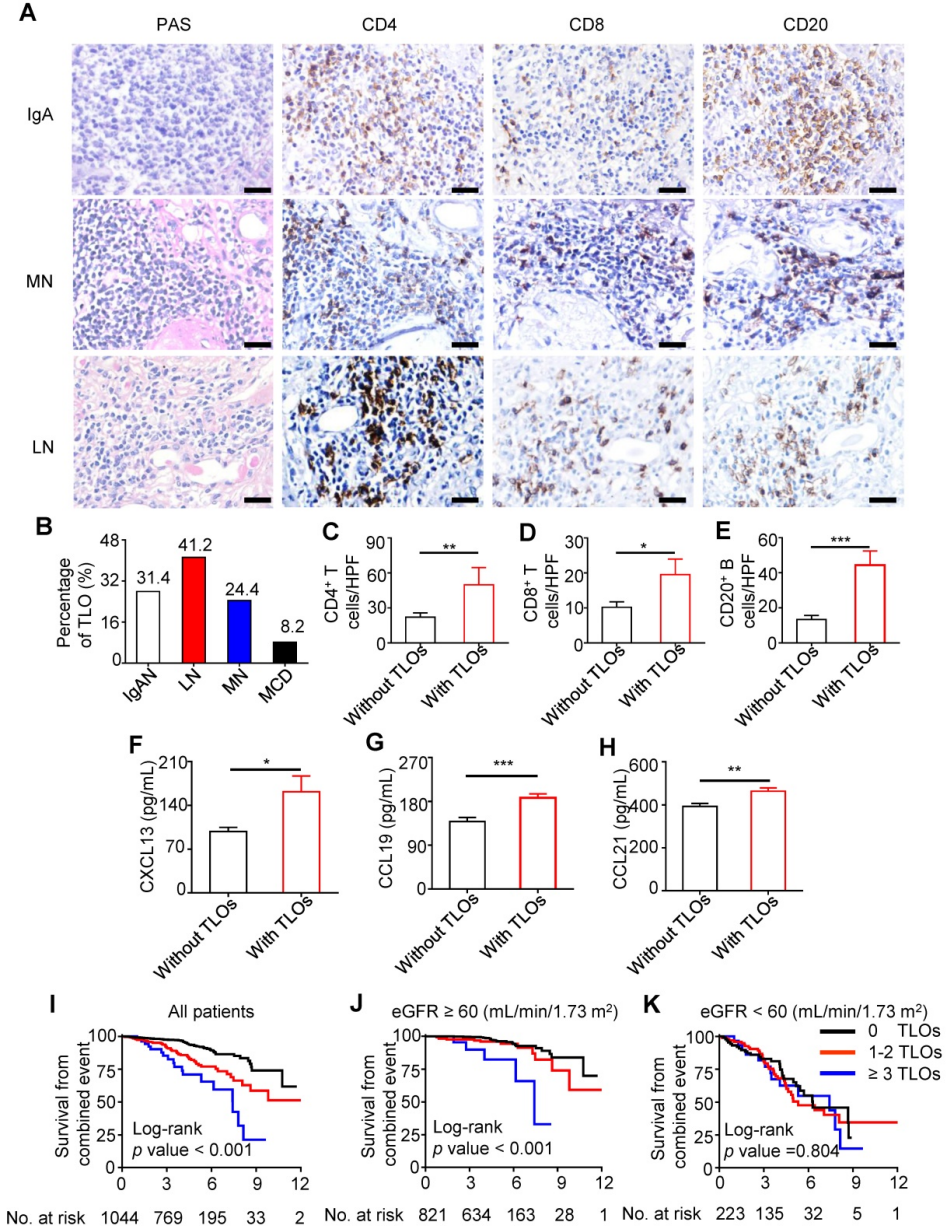
** Renal TLOs developed in patients with kidney damage and were associated with renal progression in IgAN patients.** (**A**) Representative renal TLOs in kidneys of multiple pathological types were stained by PAS. CD4^+^ T cells, CD8^+^ T cells, and CD20^+^ B cells in TLOs were stained by immunohistochemistry. (**B**) The proportion of TLOs in renal biopsy specimens of IgAN (N = 1044), LN (N = 85), MN (N = 127), and MCD (N = 49) patients. (**C-E**) Comparison of interstitial inflammatory cells, including CD4^+^ T cells (C), CD8^+^ T cells (D), and CD20^+^ B cells (E) in renal biopsy specimens of IgAN patients with TLOs group (N = 43) and without TLOs group (N = 10). (**F-H**) Plasma levels of CXCL13 (F), CCL19 (G), and CCL21 (H) were increased in IgAN patients with renal TLOs (N = 32) compared with IgAN patients without renal TLOs (N = 41). (**I-K**) Kaplan-Meier curves of the renal combined event survival of all IgAN patients (I), IgAN patients with eGFR ≥ 60 mL/min/1.73 m^2^ (J), IgAN patients with eGFR < 60 (mL/min/1.73 m^2^) (K) who were divided into three groups: without TLOs under 10 equivalent HPFs, with 1-2 TLOs under 10 equivalent HPFs, and ≥ 3 TLOs under 10 equivalent HPFs. PAS, Periodic acid-Schiff; IgAN, IgA nephropathy; LN, lupus nephritis; MN, membranous nephropathy; MCD, minimal change disease; eGFR, estimated glomerular filtration rate. *P < 0.05, **P < 0.01, ***P < 0.001. Data represent mean ± SEM, Scale bar = 20 µm. *P*-values were calculated using a two-tailed t test or Kaplan-Meier survival curves.

We investigated renal specimens of patients with kidney damage and found that TLOs were present in approximately one-third of samples. T cells and B cells were predominant in TLOs, and T cells consisted mainly of CD4^+^ T cells (Figure 1A). Among several common chronic kidney injuries, the percentage of TLOs was highest in lupus nephritis and lowest in minimal change disease (41.2% and 8.2%, respectively) (Figure 1B). Given that IgAN is the most common primary glomerulonephritis worldwide 39, we analyzed the association of TLOs formation and renal inflammation in patients with IgAN. In renal specimens, the density of CD4^+^ T cells, CD8^+^ T cells, and CD20^+^ B cells were significantly higher in patients with TLOs (Figure 1C-E). CXCL13, CCL19, and CCL21 are essential chemokines in immune cell recruitment and TLOs neogenesis. Therefore, we assessed the relationship of serum chemokines and TLOs formation in patients with IgAN. As shown in Figure 1F-H, serum CXCL13, CCL19, and CCL21 levels were significantly higher in patients with TLOs. We also assessed whether SNPs in genes related to chemokines and lymphotoxins contributed to TLOs formation. Our results showed that there was a significant difference in genotype frequency of four SNPs (rs2229094, rs1041981, rs1799964, and rs1800630) located in LTA between patients with and without TLOs (Table S2).

### The presence of renal TLOs indicated high risk of progression in IgAN

To investigate the possible association of TLOs formation and prognosis of IgAN, we enrolled 1,044 IgAN patients from the Tongji and Sun Yat-sen cohorts. The initial estimated glomerular filtration rate (eGFR) was 89.31 ± 0.98 mL/min/1.73 m^2^, and initial proteinuria was 1.09 ± 0.05 g/d. Patients were followed for a median of 4.1 (2.9, 5.5) years, during which 120 (11.5%) patients experienced a combined event (Table S3). According to the frequency of TLOs, the patients were divided into three groups: without TLOs under 10 equivalent high power fields (HPFs), with 1-2 TLOs under 10 equivalent HPFs, and ≥ 3 TLOs under 10 equivalent HPFs. Patients with more TLOs were more likely to have higher mean arterial pressure (MAP), greater proteinuria, and worse kidney function (Table S3). The score of mesangial hypercellularity (M1), proportion of segmental glomerulosclerosis present (S1), score of tubular atrophy and interstitial fibrosis (T1 and T2), and score of cellular/fibrocellular crescents (C1 and C2) were higher in patients with more TLOs (Table S4). Kaplan-Meier curves showed that survival from a combined event progressively declined as the frequency of TLOs increased; however, the association was lost in patients with baseline eGFR < 60 mL/min/1.73 m^2^ (Figure 1I-K). We analyzed the association of immunosuppressive treatment and disease progression in Tongji cohort. Of 549 patients in Tongji cohort, 416 (75.8%) received immunosuppressive treatment. Kaplan-Meier curve showed that there is no significant difference in renal survival between patients with immunosuppressive treatment and those without. When patients were grouped according to the existence of TLOs in renal biopsy, we found that there's still no significant difference in renal survival between patients with immunosuppressive treatment and those without (Figure S1). Furthermore, we performed multivariate analyses to assess the predictive value of TLOs formation. After adjustment for age, sex, MAP, proteinuria, baseline eGFR and MEST-C score, there was a graded and significant association between the frequency of TLOs and risk of combined event in all patients. Of note, the predictive value (*P* = 0.033) of TLOs formation remained significant in patients with baseline eGFR ≥ 60 mL/min/1.73 m^2^. These results suggested that TLOs can predict the progression of IgAN, especially in patients at earlier stage (Table 1).

### TLOs were induced in aged mouse kidneys

**Figure 2:**
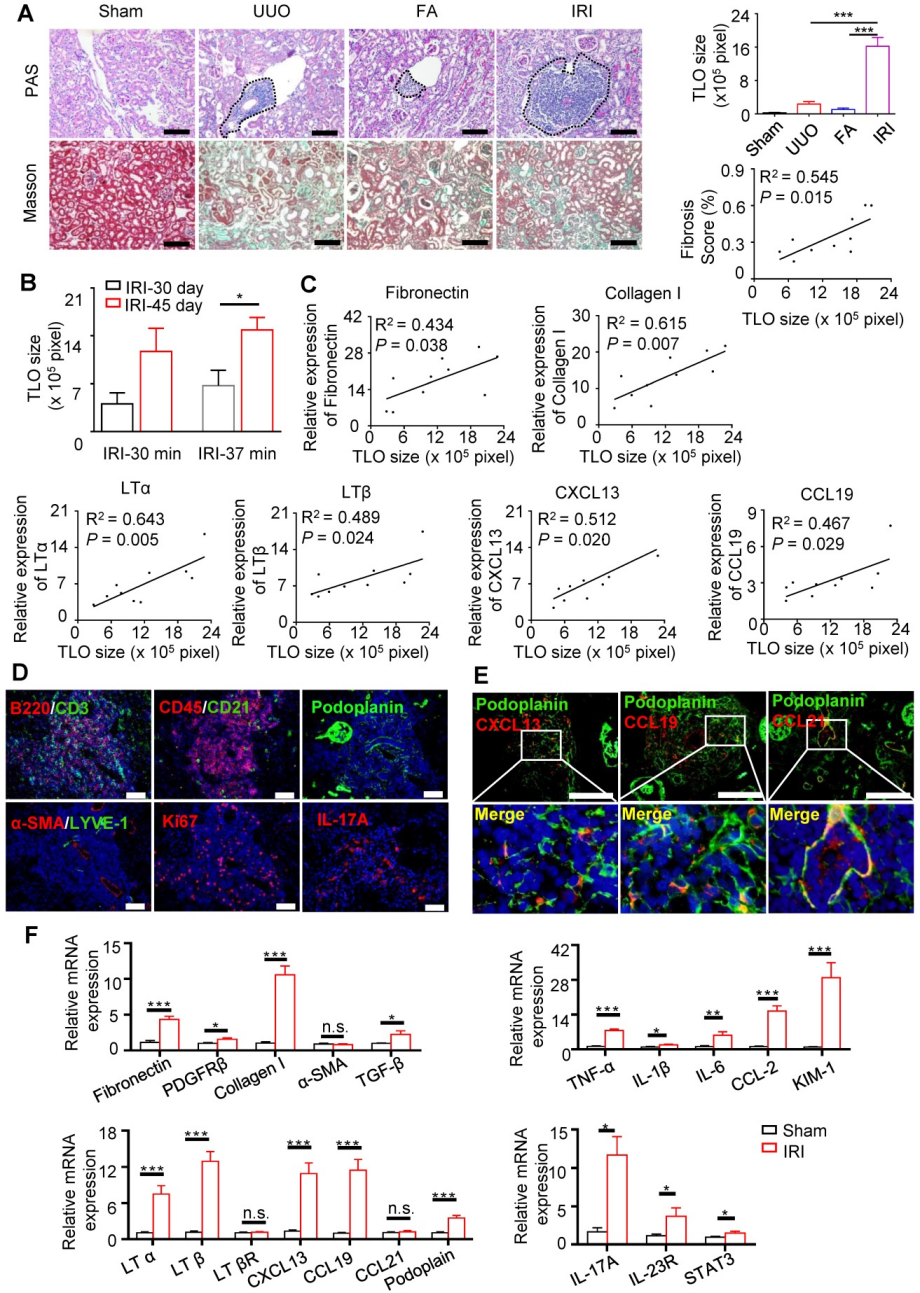
** TLOs were induced in aged mouse kidneys after renal injury.** (**A**) PAS and Masson staining. TLOs size of sham mice (N = 4), UUO mice (N = 5) (day 14), FA mice (N = 4) (day 21), and IRI mice (day 45 after 37-min IRI) (N = 6) and correlation between renal TLOs size and renal fibrosis score of mice at day 45 after 37-min IRI (N = 10). (**B**) TLOs size of mice after 30-min and 37-min IRI and sacrificed at day 30 and day 45 (N = 3 per group). (**C**) Correlation between TLOs size and mRNA level of fibronectin, collagen I, LTα, LTβ, CXCL13, and CCL19 in kidney of mice at day 45 after 37-min IRI (N = 10). (**D**) Immunofluorescence analysis of CD3 and B220, CD45 and CD21, podoplanin, LYVE-1 and αSMA, Ki67, and IL-17A. (**E**) Immunofluorescence analysis of CXCL13, CCL19 and CCL21 with podoplanin. (**F**) Renal fibrosis, inflammation, TLO-related chemokine, lymphotoxin and podoplanin, and Th17-related markers in kidneys of mice at day 45 after 37-min IRI analyzed by real-time PCR (N = 7 per group). UUO, unilateral ureteral obstruction; FA, folic acid; LYVE-1, lymphatic vessel endothelial hyaluronan receptor 1; αSMA, α-smooth muscle actin; IL-17A, Interleukin (IL)-17A. *P < 0.05, **P < 0.01, ***P < 0.001. Data represent mean ± SEM, Scale bar = 100 µm. *P*-values were calculated using a two-tailed *t* test.

We attempted to induce TLOs using mice of different ages and multiple models of kidney injury, including UUO, FA nephropathy, and unilateral IRI models. We found that TLOs formed in aged kidneys in response to kidney injury (Figure. 2A). TLOs were mostly distributed in the cortex and along the hilus of the kidney. TLOs size in the IRI model was significantly greater than in the other two kidney injury models (Figure 2A); thus, the IRI model was chosen for assessing the function and mechanism of TLOs formation. As ischemic time or the time after IRI increased, TLOs expanded and occupied larger areas of the renal parenchyma (Figure 2B). Additionally, the degree of fibrosis was correlated well with TLOs size in the IRI model (Figure 2A). Concomitantly, the expression of fibrotic markers, including fibronectin, Collagen 1 was correlated with TLOs size (Figure 2C). Moreover, the renal expression of LTα, LTβ, CXCL13, and CCL19 was also closely associated with TLOs size (Figure 2C). Thus, TLOs formed in aged kidneys after injury and could serve as a marker of sustained inflammation and renal fibrosis.

We further investigated the characterization of TLOs cellularity and structure. Numerous lymphocytes and scattered follicular dendritic cells were intermingled throughout the TLOs. It is reported that the stromal cell networks are essential for guiding lymphocyte entry and migration in secondary lymphoid organs (SLOs). We detected similar stromal cell network in TLOs, containing FRCs (podoplanin^+^), and lymphatic vessels (LYVE1^+^). TLOs also contained numerous proliferative (Ki67^+^) cells and Th17 cells (Figure 2D). Notably, the CXCL13-, CCL19-, and CCL21-positive cells within the TLOs were also positive for FRC cells (Figure 2E).

### Transcriptome analysis of kidneys in aged IRI mice

**Figure 3:**
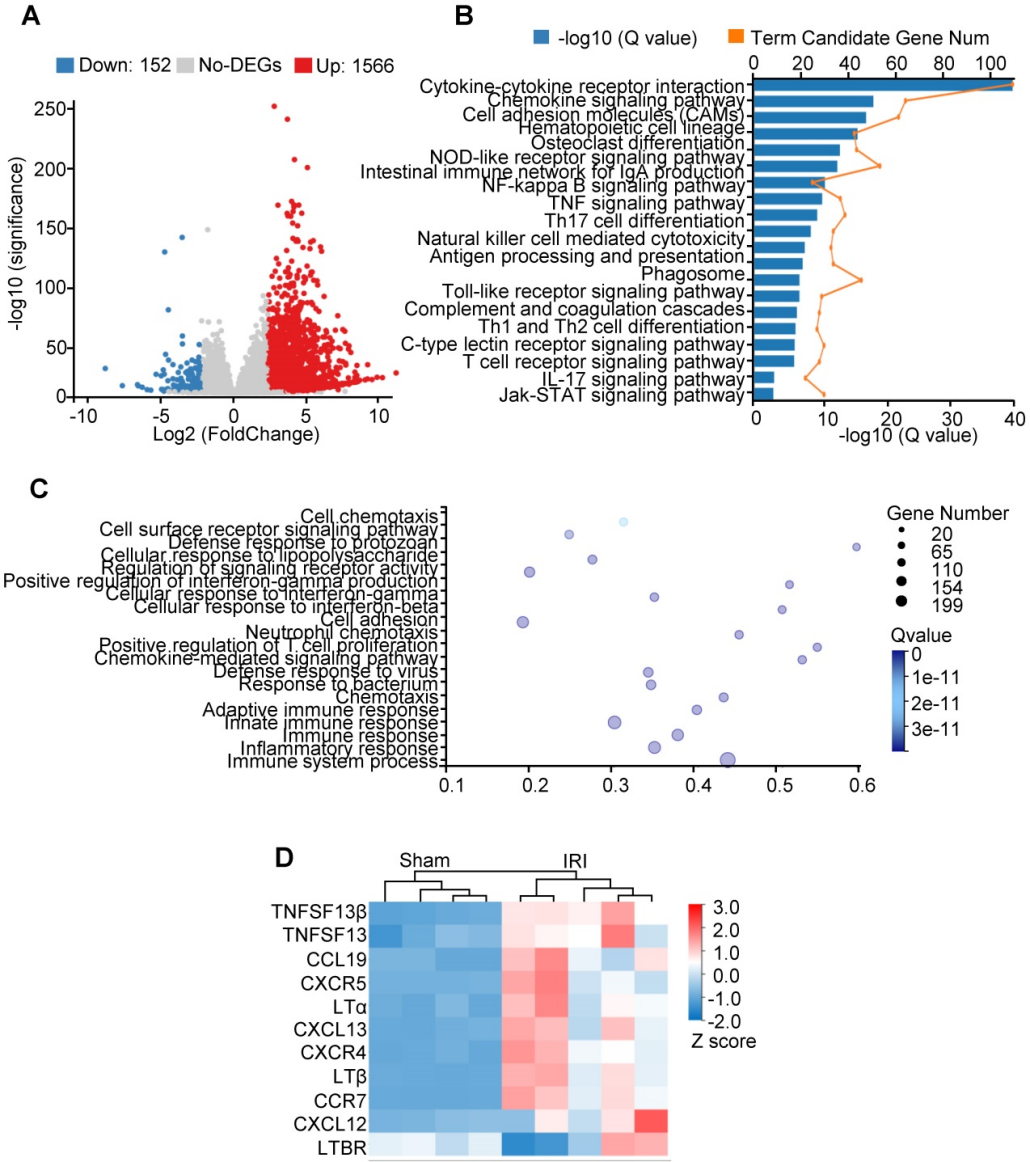
** Transcriptome analysis of kidneys in aged IRI mice.** (**A**) Volcano plot of gene expression changes. One thousand five hundred sixty-six upregulated genes and 152 downregulated genes with fold change ≥ 4 and adjusted *P* value ≤ 0.001 were found. (**B**) KEGG pathway functional enrichment analysis of all upregulated DEGs (FDR ≤ 0.01). (**C**) GO enrichment analysis of all the upregulated DEGs (FDR ≤ 0.01). (**D**) Heatmap of selected enriched terms (FDR ≤ 0.01) from KEGG pathway analysis of upregulated DEGs. GO, gene ontology; DEGs, differentially expressed genes; FDR, false discovery rate; KEGG, Kyoto Encyclopedia of Genes and Genomes.

We performed transcriptomic analysis on kidneys isolated from IRI aged mice to identify a transcriptional signature consistent with TLOs formation. Differential expression analysis was performed comparing IRI relative to the sham group, yielding a group of 1,566 significantly upregulated genes and 152 downregulated genes (Figure 3A). The KEGG pathway identified IRI kidney expression signatures associated with cytokine-cytokine receptor interaction and chemokine signaling pathway (Figure 3B). GO assessment of DEGs confirmed the activation of inflammatory response (Figure 3C). Selected TLOs formation-related genes, including Lta, Ltb, and Cxcl13, were upregulated in IRI kidneys (Figure 3D). Additionally, GO term analysis revealed significant enrichment for gene expression related to Th17 cell differentiation; further indicating Th17 cell activation within the TLOs (Figure 3B).

### Targeting TLOs formation has the potential to ameliorate renal fibrosis and inflammation

**Figure 4:**
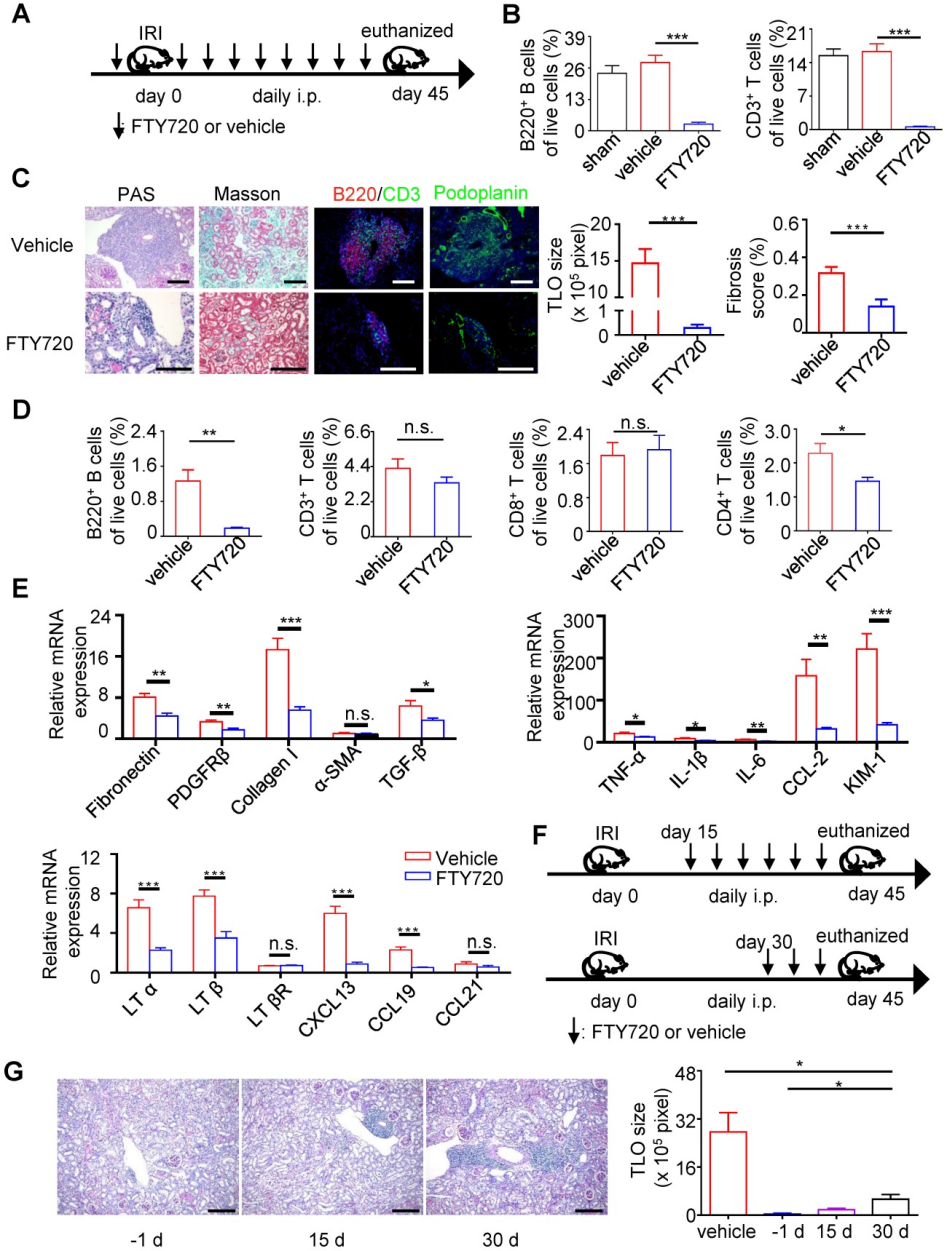
** Targeting TLOs formation has the potential to ameliorate renal fibrosis and inflammation.** (**A**) Scheme. Aged mice were treated with 0.5 mg/kg body weight FTY720 or vehicle intraperitoneally 1 day before renal IRI followed by daily injection. (**B**) The percentage of B220^+^ B cells and CD3^+^ T cells analyzed by flow cytometry from blood (N = 6 per group). (**C**) Representative photomicrograph statistical graph for PAS and Masson pathology staining and representative immunofluorescence analysis of B220 (red) with CD3 (green) and podoplanin (green). (**D**) The percentage of B220^+^ B cells, CD3^+^ T cells, CD8^+^T cells and CD4^+^ T cells was analyzed by flow cytometry from the whole kidney in the vehicle group and FTY720 treated group (N = 6 per group). (**E**) Real-time PCR analysis of renal fibrosis, TLO-related markers, and inflammation in kidneys of the vehicle group and FTY720-treated group (N = 6 per group). (**F**) Scheme. Aged mice were treated with 0.5 mg/kg body weight FTY720 or vehicle intraperitoneally at day 15 or day 30 after renal IRI followed by daily injection. (**G**) Representative photomicrographs and statistical graph for PAS staining of the FTY720 treatment group starting from day -1, day 15, and day 30 (N = 3 per group). *P < 0.05, **P < 0.01, ***P < 0.001. Data represent mean ± SEM, Scale bar = 100 µm. *P*-values were calculated using a two-tailed *t* test.

FTY720 (fingolimod) is a modulator of sphingosine-1-phosphate receptors. It can block lymphocyte egress from lymph nodes and consequently limit their recruitment 40. FTY720 has been reported to potently inhibit the accumulation of lymphocytes in inflamed lymphoid organ and tissues 41. To assess the contribution of TLOs in kidney injury, we used FTY720 to prevent the formation of renal TLOs after kidney injury in aged mice (Figure 4A). Application of FTY720 led to a sharp decline of blood B cells and T cells, indicating that lymphocyte recirculation was largely prevented (Figure 4B). As expected, TLOs were significantly smaller in FTY720-treated kidneys, although TLOs had similar cellular components (Figure 4C). Flow cytometry showed that the percentage of B220^+^ B cells was reduced significantly in FTY720-treated kidney. The percentage of CD3^+^ T cell also showed a descending tendency. Moreover, the percentage of CD4^+^ T cells declined significantly, while the percentage of CD8^+^ T cells was not altered (Figure 4D). Notably, FTY720 significantly attenuated renal fibrosis and reduced the levels of fibrosis markers and inflammation cytokines (Figure 4C, E). The expression of LTα, LTβ, CXCL13, and CCL19 were also reduced in FTY720-treated kidneys (Figure 4E). To evaluate whether the administration of FTY720 in the later stage of kidney injury course could prevent TLOs formation, we administrated FTY720 at 15 days or 30 days after IRI and then euthanized the mice at 45 days (Figure 4F). Our result showed that the renal TLOs size was greater in mice with FTY720 treatment after IRI than before IRI. Moreover, the effect of FTY720 treatment started at 30 days was not as prominent as that at 15 days (Figure 4G-H). Therefore, early intervention may be necessary to prevent TLOs formation.

### IL-17A is needed in renal TLOs development

**Figure 5:**
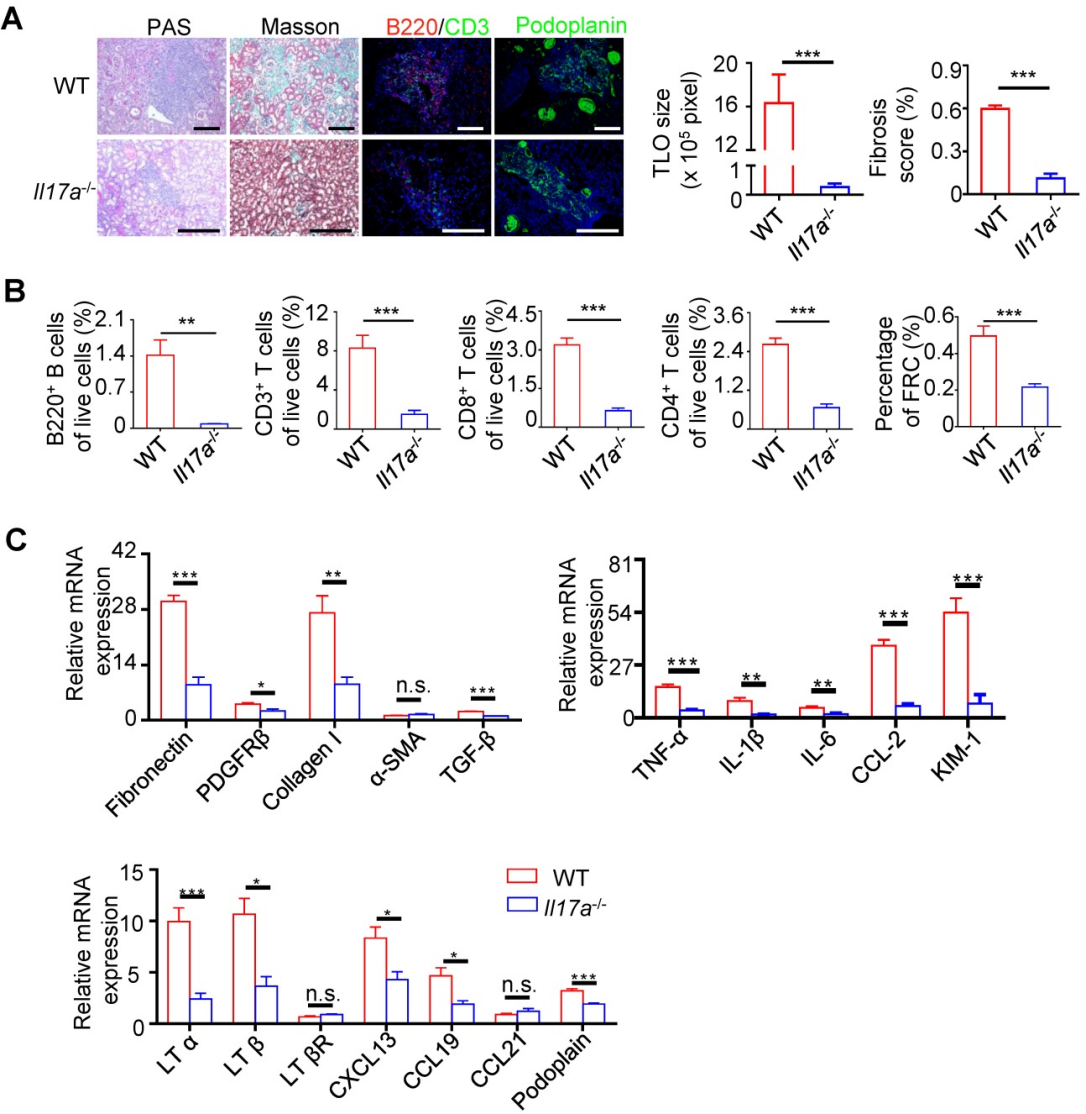
** The development of TLOs required IL-17A.** (**A**) Representative photomicrograph statistical graph for PAS and Masson pathology staining and representative immunofluorescence analysis of B220 (red) with CD3 (green) and podoplanin (green) in the WT and *Il17a^-/-^* groups. (**B**) The percentage of B220^+^ B cells, CD3^+^ T cells, CD8^+^ T cells, CD4^+^ T cells, and CD31^-^GP38^+^ FRCs was analyzed by flow cytometry from the whole kidneys in the WT and *Il17a^-/-^* groups (N = 6 per group). (**C**) Real-time PCR analysis of renal fibrosis, inflammation, and TLO-related markers in kidneys of the WT and *Il17a^-/-^* groups (N = 6 per group). *P < 0.05, **P < 0.01, ***P < 0.001. Data represent mean ± SEM, Scale bar = 100 µm. *P*-values were calculated using a two-tailed *t* test.

**Figure 6:**
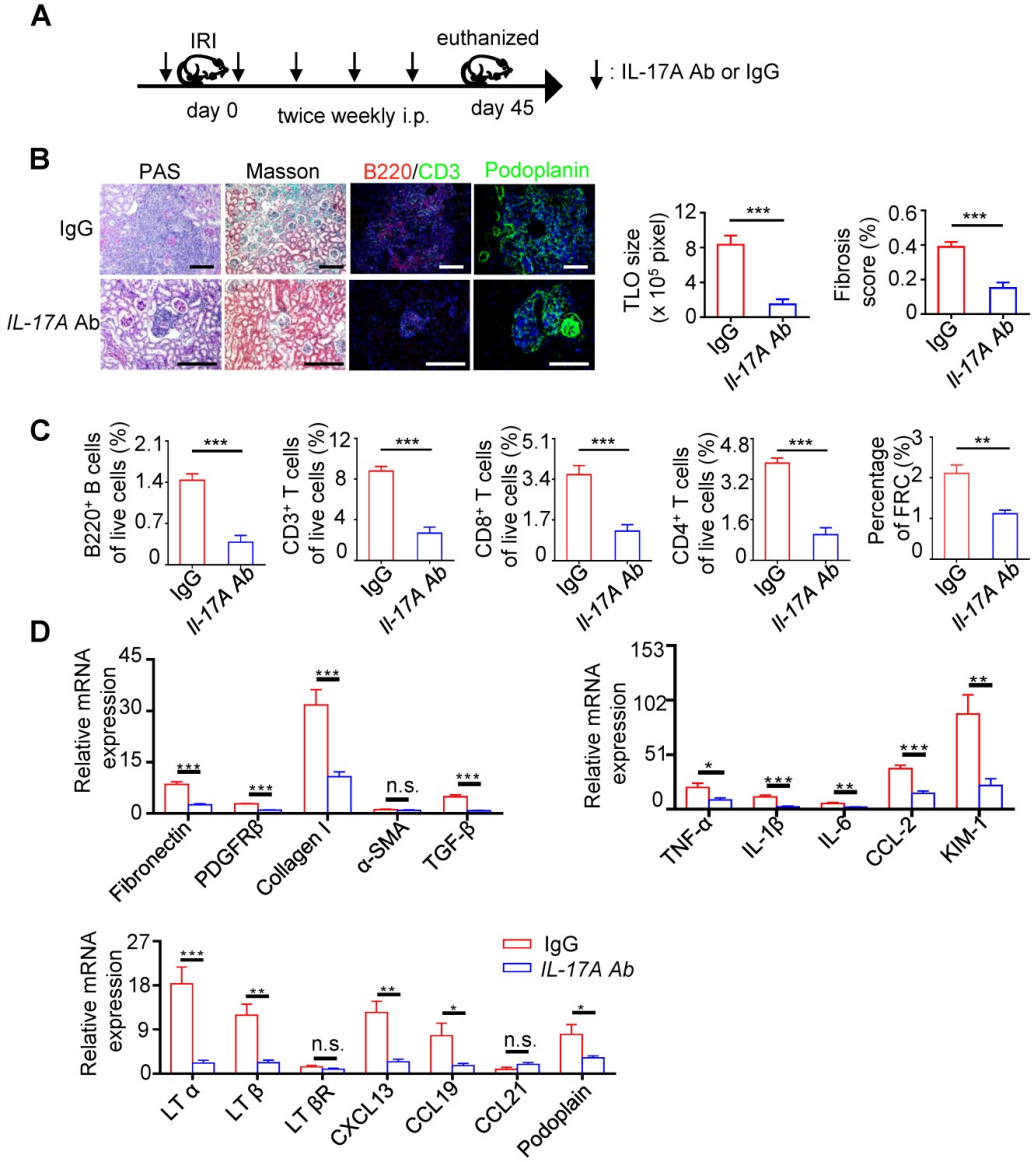
** Neutralization of IL-17A inhibited TLOs formation and alleviated renal fibrosis and inflammation of aged mice after IRI.** (**A**) Scheme. Aged mice were injected intraperitoneally with either 50 µg of anti-IL-17A antibody or IgG at 1 day before renal IRI followed by daily injection (IgG group N = 5, IL-17A Ab N = 6). (**B**) Representative photomicrograph of pathology staining Masson pathology staining and representative immunofluorescence analysis of B220 (red) with CD3 (green) and podoplanin (green) in the IgG and IL-17A Ab groups. (**C**) The percentage of B220^+^ B cells, CD3^+^ T cells, CD8^+^ T cells, CD4^+^ T cells, and CD31^-^GP38^+^ FRCs were analyzed by flow cytometry from whole kidneys in the IgG and IL-17A Ab groups (IgG group N = 5, IL-17A Ab N = 6). (**D**) Real-time PCR analysis of renal fibrosis, inflammation, and TLO-related markers in kidneys of the vehicle and IL-17A Ab groups (IgG group N = 5, IL-17A Ab N = 6). *P < 0.05, **P < 0.01, ***P < 0.001. Data represent mean ± SEM, Scale bar = 100 µm. *P*-values were calculated using a two-tailed *t* test.

Th17 cells share multiple similar properties with LTi cells and are reported to be involved in the development of TLOs. We found that the expression of IL-17A, IL23R, and STAR3 increased significantly in IRI kidneys (Figure 2F). In addition, our transcriptome analysis indicated that DEGs were enriched in Th17 cell differentiation (Figure 3B). To determine the role of IL-17A in the development of renal TLOs, we evaluated whether the lack of IL-17A affects TLOs formation. We found that IRI-induced TLOs were smaller in *Il17a^-/-^* mice, and renal fibrosis was alleviated (Figure 5A). TLOs in *Il17a^-/-^* mice had similar cellular components, containing B lymphocytes, T lymphocytes, and FRC networks (Figure 5A). We next tested whether the percentage of these TLOs component cells changed in *Il17a^-/-^* mice using flow cytometry. We found that the percentage of B220^+^ B cells, CD3^+^ T cells, CD4^+^T cells, CD8^+^ T cells, and FRCs in all live cells was remarkably reduced in *Il17a^-/-^* mice (Figure 5B). Moreover, the expression of proinflammatory cytokine, and fibrosis markers was significantly decreased in *Il17a^-/-^* mice (Figure 5C). The expression of homeostatic chemokines, including LTα, LTβ, CXCL13, and CCL19, was also reduced (Figure 5C). We further examined whether treatment with the IL-17A antibody prevented TLOs development. We therefore administered IL-17A antibody twice weekly by intraperitoneal injection after ischemic insults (Figure 6A) and found that TLOs formation was abolished and renal fibrosis was attenuated (Figure 6B). The lymphocytes and FRCs in kidneys were also significantly reduced (Figure 6C). Moreover, the expression of fibrosis markers, proinflammatory cytokines and homeostatic chemokines was decreased (Figure 6D). This suggested that anti-IL17A therapy may have the therapeutic potential to improve renal outcomes by suppressing TLO-mediated inflammation.

## Discussion

In the present study, we showed that renal TLOs existed in various chronic kidney injuries, including IgAN, lupus nephritis, and membranous nephropathy. Furthermore, in a large multicenter prospective cohort, we demonstrated that the frequency of renal TLOs was closely correlated with the progression of IgAN. TLOs developed within the kidneys in aged mice after ischemia reperfusion injury. After inducing lymphopenia by FTY720, the formation of renal TLOs was inhibited. Together with the evidence that *Il17a^-/-^* aged mice had smaller TLOs after renal ischemia reperfusion injury, the administration of blocking antibodies to IL-17A also reduced the development of TLOs correlating with a modest reduction in renal fibrosis.

We observed the renal TLOs in various patients with kidney damage, which indicated that renal TLOs were a common pathologic feature of chronic kidney injury. In addition, renal TLOs can be clearly recognized in PAS stained sections, thus, it is easy to apply in practice. In our study, renal TLOs were defined as aggregates of T cells and B cells. However, unlike TLOs in tumor 42 and adventitia 43, most of the renal TLOs did not exhibit structural segregation of T and B cell zones. One possible reason is the small kidney biopsy size; thus, matured stages of TLOs are rarely observed in limited renal TLOs. It has been reported that TLOs aggravate disease course and proliferate chronic autoimmune response in some chronic inflammation disease 44,45. We found that renal TLOs were correlated with more interstitial inflammatory cells in IgAN, which is consistent with our previous study with small sample sizes. This invited speculation about the role of TLOs in aggravating intrarenal inflammation. More studies are needed to evaluate the mechanism between renal TLOs with intrarenal inflammation and immunity.

This study demonstrated the correlation of renal TLOs and disease progression in patients with IgAN. TLOs have been reported to promote antigen-specific responses that improve anti-tumor and anti-pathogen immunity in certain cancers and infections 46. In contrast, in autoimmune diseases, TLOs can reinforce local autoantibody responses and exacerbate diseases 47. However, it is unclear whether TLOs provide pathogenic or protective contributions to kidney diseases. In our study, we found that IgAN patients with ≥ 3 TLOs under 10 equivalent HPFs tended to have poor prognosis after 4.1 years of follow-up. More intriguingly, renal TLOs were an independent risk factor for IgAN patients after multivariate adjustment. These results suggested that TLOs contributed to pathogenesis and had potential prediction ability for disease progression in IgAN. Thus, renal TLOs may be considered as a histological indicator when assessing renal specimens of IgAN in clinical routine. Notably, in subgroup analysis, for patients with eGFR more than 60 mL/min/1.73 m^2^, TLOs were an independent risk factor after adjustment. Conversely, for patients with eGFR less than 60 mL/min/1.73 m^2^, TLOs were not related with disease progression. According to these findings, we speculated that renal TLOs may play an important role in the pathological process of kidney injury at early stage, and as kidney injury continues to advance, renal TLOs seem less crucial.

The mechanism of TLOs formation and regulation in other organs has been well studied; however, renal TLOs formation has been rarely reported. In this study, we established IRI, UUO, and FA renal injury models of aged mice to induce renal TLOs. We found that the size of TLOs in the IRI model were greater than in the UUO and FA renal injury models, which may be due to the different processes of injury model establishment. Because TLOs need considerable time to form and the UUO model and the FA model were sacrificed at day 14 and day 21, while mice in the IRI model were sacrificed at day 45. We speculated that the short duration may result in small TLOs size. Interestingly, TLOs size was positively correlated with renal fibrosis. Tatsuaki Watanabe also observed that lung graft fibrosis was correlated with TLOs content 48. Therefore, further investigation the potential mechanisms by which TLOs may promote renal fibrosis is warranted. In addition, in aged injured mice, renal TLOs were mainly found adjacent to vessels, which is consistent with those observed in renal specimens of patients with kidney damage. These suggested that circulating lymphocytes may migrate from these vessels, thereby accumulating and proliferating locally.

FRCs play a critical role in the trafficking of lymphocytes into the SLOs by secreting chemokines 49,50. In our study, we found that FRCs were increased in aged kidneys of IRI model mice and served as an important source of chemokines, including CXCL13 and CCL19. Moreover, the expression level of chemokines was correlated with renal TLOs size. These results suggested that FRCs and chemokines may also contribute to the initiation of TLOs formation. Remarkably, we found that plasma levels of CXCL13, CCL21, and CCL19 were significantly higher in patients with renal TLOs, indicating a possible correlation between abnormally increased plasma chemokine levels and TLOs formation in IgAN patients. Circulating chemokines may reflect renal TLOs formation as a non-invasive biomarker. Thus, additional studies are needed to investigate the predictive value of plasma chemokine levels in disease progression and treatment response of patients with IgAN. Recently, studies have shown that plt/plt mice that lack the CCL19 and CCL21 were resistant to TLOs formation in the EAE model 51. It has been reported that neutralization of CXCL13 partially protected mice against cigarette smoke-induced lymphoid follicle formation 52, and depletion of CXCL13 also inhibited IRI-induced renal TLOs development 53. Blocking the biological effects of chemokines might be an important treatment target of patients with IgAN in the future.

FTY720 acts on lymphocyte sphingosine-1-phosphate 1 receptors and induces long-lasting lymphopenia in numerous models, such as multiple sclerosis and diabetes 41,54. Our results showed that FTY720 diminished TLOs formation in kidneys and decreased TLO-related chemokine CXCL13 and CCL19 expression, which is consistent with previous animal research on TLOs in EAE and diabetes. FTY720 was approved by the United States Food and Drug Administration as an oral first-line treatment for relapsing-remitting multiple sclerosis 55. We found that FTY720 significantly reduced the expression of proinflammatory cytokines and fibrosis markers. Therefore, FTY720 may have potential as a therapy method to alleviate renal inflammation and fibrosis, which needs to be verified in further clinical trials. In addition, we showed that late administration of FTY720 after renal IR surgery could not completely halt TLOs formation compared with early administration of FTY720 at the start of renal injury. Notably, our clinical analysis demonstrated that TLOs were independent risk factors of disease progression at early stage of IgAN. Thus, early intervention of patients with renal TLOs should be emphasized.

IL-17A has been reported to be a vital inflammatory factor and involved in several different kidney diseases 56-58. Additionally, circulating IL-17A levels were increased in patients with chronic kidney disease 17. Blocking IL-17RA present on the surface of bone marrow cells could relieve renal fibrosis 59. In our study, IL-17A was obviously increased in renal TLOs models. Moreover, we found that renal TLOs induced in aged *Il17a^-/-^* mice were smaller compared with wild-type aged mice, this finding was consistent with other disease models focused on TLOs regulated by IL-17A, such as experimental autoimmune encephalitis 25 and lipopolysaccharide-induced pulmonary inflammation 26. Meanwhile, the expression of TLO-related chemokines CXCL13 and CCL19 decreased after IL-17A depletion. In our study, FRCs were found to be an important source of these chemokines, and the number of renal FRC cells was also decreased in the *Il17a^-/-^* IRI model compared with wild-type. IL-17A was reported to potentiate FRCs proliferation and survival 60. These findings provided insight into the involvement of IL-17A in renal TLOs formation by promoting FRCs network development and chemokine secretion. In addition, we found that the administration of IL-17A antibody also inhibited TLOs formation. Thus, IL-17A could be a therapy target for kidney damage, which needs more clinical evidence.

The current study has several limitations. First, our cohort is a retrospective study; a prospective study is required to further confirm the predictive effect of TLOs on renal progression of kidney damage. Second, we studied the blocking effect of FTY720 on lymphocyte circulation, but whether the effect on stromal cells would affect the formation of TLOs was not further discussed.

In conclusion, this study provides evidence that renal TLOs were correlated with disease severity and had potent prediction ability for the progression of kidney damage, especially at early stage of diseases. Plasma levels of chemokines that were correlated with renal TLOs might predict renal prognosis as non-invasive biomarkers. Moreover, IL-17A may be involved in the formation of renal TLOs through promoting the FRCs network development and chemokine secretion. Thus, our insight into renal TLOs formation and regulation provide new approaches and targets for the treatment of kidney damage.

## Supplementary Material

Supplementary figures and tables.

## Figures and Tables

**Table 1:** Cox regression analyses of renal TLOs with combined event in patients with IgA nephropathy

		All patients (N = 1044)		eGFR < 60 mL/min/1.73m^2^ (N = 223)		eGFR ≥ 60 mL/min/1.73m^2^ (N = 821)	
		HR (95% CI)	*P* value	HR (95% CI)	*P* value	HR (95% CI)	*P* value
Unadjusted	1-2 TLO	2.58 (1.75, 3.80)	< 0.001	1.14 (0.70, 1.84)	0.607	1.80 (0.89, 3.61)	0.101
	≥ 3 TLO	5.31 (3.10, 9.09)	< 0.001	1.22 (0.63, 2.35)	0.556	7.72 (2.91, 20.5)	< 0.001
Adjusted	1-2 TLO	1.26 (0.82, 1.96)	0.291	1.05 (0.60, 1.85)	0.856	1.71 (0.79, 3.71)	0.173
	≥ 3 TLO	1.38 (0.75, 2.56)	0.303	0.86 (0.40, 1.84)	0.696	3.44 (1.11, 10.74)	0.033

HR, hazard ratio; 95% CI, 95% confidence interval; TLO, tertiary lymphoid organs. Multivariable analysis was adjusted for age, sex, mean arterial pressure, estimate glomerular filtration rate (eGFR), proteinuria and MEST-C score. Combined event was defined as 50% decline of eGFR or end-stage renal disease.

## Abbreviations

DAB: 3,3'-diaminobenzidine
DAPI: 4′,6-diamidino-2-phenylindole
DEGs: differentially expressed genes
EAE: experimental autoimmune encephalomyelitis
eGFR: estimated glomerular filtration rate
ESRD: end-stage renal disease
FA: folic acid
FRCs: fibroblastic reticular cells
FPKM: fragments per kilobase million
GO: gene ontology
HEVs: high endothelial venules
HRP: horseradish peroxidase
HPFs: high power fields
HISAT: Hierarchical Indexing for Spliced Alignment of Transcripts
IL-17A: interleukin-17A
IRI: ischemic reperfusion injury
KEGG: Kyoto Encyclopedia of Genes and Genomes
IgAN: IgA nephropathy
LTi: lymphoid tissue inducer
LTo: lymphoid tissue organizing
LN: lupus nephritis
LV: lymphatic vessels
MAP: mean arterial pressure
MCD: minimal change disease
MN: membranous nephropathy
PAS: Periodic acid-Schiff
PBS: phosphate-buffered saline
PCR: polymerase chain reaction
SNP: single-nucleotide polymorphism
SLOs: secondary lymphoid organs
TLOs: Tertiary lymphoid organs
UUO: unilateral ureteral obstruction
