# Supplementary material for: Tertiary lymphoid organs are associated with the progression of kidney damage and regulated by interleukin-17A
Source: Theranostics. 2021 Jan 1;11(1):117–31. doi: 10.7150/thno.48624 (PMC7681089; doi:10.7150/thno.48624)
Supplement: Supplementary file 1 — Supplementary figures and tables. [file thnov11p0117s1.pdf]

Table S1. Primers used to amplify cDNA for mice.

Primers	Sequence (Sense/Antisense)
Fibronectin	5′-ATGATGAGGTGCACGTGTGT-3′
	5′-TCTCCCAGGAGTCACCAATC-3′
TGF-β	5′-CGCAACAACGCCATCTATGA-3′
	5′-ACCAAGGTAACGCCAGGAAT-3′
Collagen-1	5′-GTCCTAGTCGATGGCTGCTC-3′
	5′-CAATGTCCAGAGGTGCAATG-3′
α-SMA	5′-GGAGAAGCCCAGCCAGTCGC-3′
	5′-AGCCGGCCTTACAGAGCCCA-3′
PDGFR-β	5′-GGGTCCGTTCCAGAAAATGT-3′
	5′-CAAGGGACCGGGGTCCAA-3′
IL-1β	5′-GAAATGCCACCTTTTGACAGTG-3′
	5′-TGGATGCTCTCATCAGGACAG-3′
IL-6	5′-CTGCAAGAGACTTCCATCCAG-3′
	5′-AGTGGTATAGACAGGTCTGTTGG-3′
CCL-2	5′-TAAAAACCTGGATCGGAACCAAA-3′
	5′-GCATTAGCTTCAGATTTACGGGT-3′
KIM-1	5′-TAAACCAGAGATTCCCACAC-3′
	5′-GATCTTGTTGAAATAGTCGTGG-3′
TNF-α	5′-CCTGTA GCCCACGTCGTAG-3′
	5′-GGGAGTAGACAAGGTACAACCC-3′
LTα	5′-CCCATCCACTCCCTCAGAAG-3′
	5′-CATGTCGGAGAAAGGCACGAT-3′
LTβ	5′-GAGACAGTCACACCTGTTG-3′
	5′-CCTGTAGTCCACCATGTCG-3′
LTβR	5′-TGGTGCTCATCCCTACCTTC-3′
	5′-TCCCAAACTCTCCTCCACAC-3′
CXCL13	5′-CGTGCCAAATGGTTACAAAGATT-3′
	5′-GTGGCTTCAGGCAGATCTTC-3′
CCL19	5′-CCTGGGAACATCGTGAAAGC-3′
	5′-TGGAGGTGCACAGAGCTGATA-3′
CCL21	5′-AAGGCAGTGATGGAGGGG-3′
	5′-CGGGGTAAGAACAGGATTG-3′
IL-17A	5′-AAGGCCCTCAGACTACCTCAAC-3′
	5′-TGAGCTTCCCAGATCACAGAG-3′
Podoplanin	5′-TCCGATGAGTTGAGGAGCCA-3′
	5′-AGAAGCAGAAGGCAGGTGTTAGA-3′
β-actin	5′-AGAGGGAAATCGTGCGTGAC-3′
	5′-CAATAGTGATGACCTGGCCGT-3′
Stat3	5′-CAGTTTGAGTCGCTCACGTTTG-3′
	5′-TCTCTGCAGCTTCTGGTTTCA-3′
IL-23R	5′-AACAACAGCTCGGATTTGGTAT-3′

	5′-ATGACCAGGACATTCAGCAGT-3′

Table S2. Genotype frequencies for chemokines and lymphotoxin genes in patients with renal TLOs versus those without.

Gene	dbSNP	Genotype	With TLOs	Without TLOs	P value
		CC	286	494	
LTA	rs2239704	TC	37	69	0.614
		TT	4	1	
		AA	88	144	
LTA	rs909253	GA	150	303	0.053
		GG	92	124	
		TT	242	362	
LTA	rs2229094	CT	75	193	**0.002**
		CC	13	16	
		CC	88	144	
LTA	rs1041981	AC	150	304	**0.045**
		AA	92	123	
		TT	243	365	
LTA	rs1799964	CT	74	190	**0.003**
		CC	12	16	
		CC	254	386	
LTA	rs1800630	AC	66	169	**0.006**
		AA	10	14	
		CC	224	413	
LTA	rs1799724	CT	92	147	0.269
		TT	11	11	
		CC	114	197	
LTBR	rs3759333	TC	160	260	0.500
		TT	56	114	
		GG	273	489	
CCL19	rs2227302	AG	47	69	0.583
		AA	3	4	
		AA	284	474	
CCL19	rs2233872	GA	40	85	0.372
		GG	1	4	
		AA	289	502	
CCL21	rs2812378	GA	36	63	0.987
		GG	2	3	
		AA	160	281	
CCL21	rs2812377	CA	122	211	0.852
		CC	29	44	

Table S3. Baseline characteristics of overall study cohort and according to the frequency of renal TLOs

	All patients	Without TLO	With 1-2 TLOs	With ≥ 3 TLOs	P value
Number	1044	716	273	55	
Age, years	33.67 ± 0.31	33.24 ± 0.39	34.54 ± 0.59	34.89 ± 1.37	0.131
Sex (Male, %)	40.9	38.5	46.5	43.6	0.068
MAP, mmHg	95.91 ± 0.45	94.50 ± 0.52	98.56 ± 0.88	101.00 ± 2.35	< 0.001
Hemoglobin, g/L	127.70 ± 0.59	129.20 ± 0.69	125.50 ± 1.15	119.00 ± 3.44	< 0.001
Albumin, g/L	39.33 ± 0.17	39.77 ± 0.21	38.73 ± 0.32	36.66 ± 0.79	< 0.001
Uric acid, μmol/L	353.20 ± 3.52	338.80 ± 3.98	378.90 ± 7.36	417.80 ± 16.27	< 0.001
Proteinuria, g/d	1.09 ± 0.05	1.01 ± 0.06	1.15 ± 0.08	1.69 ± 0.23	0.002
eGFR, mL/min/1.73 m^2	89.31 ± 0.98	96.04 ± 1.07	77.92 ± 1.95	58.22 ± 3.92	< 0.001
Length of follow-up, years	4.1 (2.9, 5.5)	4.2 (3.0, 5.6)	4.1 (2.8, 5.3)	3.5 (2.2, 4.9)	0.129
Combined event, %	11.5	7.3	18.3	32.7	< 0.001
eGFR at last follow-up, mL/min/1.73 m^2	81.45 ± 35.12	88.61 ± 31.77	69.93 ± 37.45	54.39 ± 33.12	< 0.001

TLO, tertiary lymphoid organs; MAP, mean arterial pressure; eGFR, estimated glomerular rate.
According to the frequency of TLOs, the patients were divided into three groups: without TLOs under 10 equivalent HPFs, with 1-2 TLOs under 10 equivalent HPFs, and ≥ 3 TLOs under 10 equivalent HPFs.
Combined event was defined as 50% decline of eGFR or end-stage renal disease.

Table S4. The correlation between MEST-C score of overall study cohort and the frequency of renal TLOs

MEST-C score	All patients (N = 1044)	Without TLO (N = 716)	With 1-2 TLOs (N = 273)	With ≥3 TLOs (N = 55)	P value
Mesangial hypercellularity					0.005
score ≤ 0.5 (M0)	551 (52.8%)	399 (55.7%)	132 (48.4%)	20 (36.4%)	
score > 0.5 (M1)	493 (47.2%)	317 (44.3%)	141 (51.6%)	35 (63.6%)	
Endocapillary hypercellularity					0.165
Absent (E0)	627 (60.0%)	425 (59.4%)	174 (63.7%)	28 (50.9%)	
Present (E1)	417 (39.9%)	291 (40.6%)	99 (36.3%)	27 (49.1%)	
Segmental glomerulosclerosis					< 0.001
Absent (S0)	416 (39.8%)	318 (44.4%)	84 (30.8%)	14 (25.5%)	
Present (S1)	628 (60.1%)	398 (55.6%)	189 (69.2%)	41 (74.5%)	
Tubular atrophy and interstitial fibrosis					< 0.001
≤ 25% (T0)	707 (67.7%)	565 (78.9%)	127 (46.5%)	15 (27.3%)	
26% to 50% (T1)	254 (24.3%)	119 (16.6%)	108 (39.6%)	27 (49.1%)	
> 50% (T2)	83 (7.9%)	32 (4.5%)	38 (13.9%)	13 (23.6%)	
Cellular/fibrocellular crescents					0.001
0% (C0)	631 (60.4%)	454 (63.4%)	155 (56.8%)	22 (40.0%)	

1% to 25% (C1)	375 (35.9%)	243 (33.9%)	102 (37.4%)	30 (54.5%)
> 25% (C2)	38 (3.6%)	19 (2.7%)	16 (5.8%)	3 (5.5%)

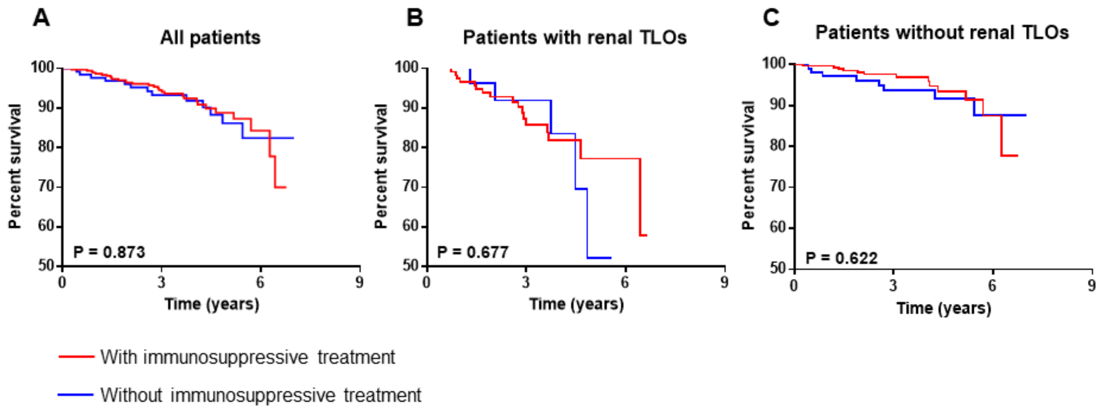

Figure S1. Prognosis of IgAN patients was not associated with immunosuppressive treatment

Kaplan-Meier curves of the renal combined event survival of IgAN patients in Tongji cohort divided by immunosuppressive treatment in all patients (A), in patients with renal TLOs (B), in patients without renal TLOs (C).
